# Medical students' perspectives on biomedical informatics learning objectives

**DOI:** 10.5116/ijme.50ce.316b

**Published:** 2013-01-11

**Authors:** Denise E. Beaudoin, Stephanie J. Richardson, Xiaoming Sheng, Joyce A. Mitchell

**Affiliations:** 1 Department of Biomedical Informatics, University of Utah, USA; 2 Division of Acute and Chronic Care, College of Nursing, University of Utah, USA; 3 Pediatric Research Enterprise, University of Utah, USA

**Keywords:** Biomedical informatics, undergraduate medical education, competency-based education, curriculum development, integrated curriculum

## Abstract

Objectives: To explore medical student perspectives regarding the importance of biomedical informatics learning objectives to career development, and the amount of emphasis that should be placed on content associated with these objectives in the curriculum.

**Methods:**

A Web-based survey was e-mailed to 405 students enrolled at the University of Utah, School of Medicine in spring 2008. Respondents rated the importance of biomedical informatics learning objectives using a five-point Likert-type scale, and indicated whether this content should be given a minimal, moderate or large amount of emphasis. ANOVA and the Kruskal-Wallis test were conducted to determine differences in perceived importance and desired emphasis by academic year.

**Results:**

A total of 259 medical students submitted a survey for an overall response rate of 63.9%. Learning objectives associated with the physician role of Clinician received the highest overall rating (mean = 3.29 ± 0.47). Objectives for the physician roles of Clinician, Life-long Learner and Manager received higher ratings than the Educator/Communicator and Researcher roles in terms of both perceived importance and amount of emphasis. Student ratings of importance varied significantly by academic year, with third-year students consistently assigning lower ratings to learning objectives for the Educator/Communicator, Researcher and Manager roles compared to students in some other years.

**Conclusions:**

Study results suggest that biomedical informatics content is desired by medical students at the University of Utah. Study findings are being used to inform efforts to integrate biomedical informatics content into the curriculum and may assist other medical schools seeking to incorporate similar content.

## Introduction

Given the expanding role of technology and information management in today’s health care environment, it is critical that physicians-in-training become proficient in biomedical informatics knowledge and skills.^1^^-^^3^ Informatics competencies focusing on the educational needs of clinicians and health information management specialists have been defined by several professional organizations.^4^^-^^6^ The Association of American Medical Colleges (AAMC) has recommended that medical students also receive a foundation in informatics. The Medical School Objectives Project (MSOP) is an AAMC initiative designed to reach consensus on the skills, attitudes and knowledge that medical students should possess by the time of graduation.^7^ An MSOP Advisory Panel convened in 1998 identified a set of informatics learning objectives for medical students, and recommended that these be integrated into medical school curricula.^7^ More recently, scientific competencies related to informatics have been defined for future medical school graduates.^8^

In a 2007-2008 survey, 108 of 126 US medical schools (86%) reported that informatics was included as a required course.^9^ However, according to a survey conducted in 2006, only a few medical schools taught MSOP learning objectives that required interaction with health information.^1^ The integration of biomedical informatics competencies into existing curricula presents both a challenge and an opportunity for novel educational research. Previous surveys of medical students have assessed their technology needs and perceived computer and informatics competencies.^10^^-^^13^ However, to our knowledge, no study has queried physicians-in-training about the importance of informatics learning objectives in terms of role development. The objectives of the present study were to determine medical student perceptions regarding the importance of the MSOP informatics learning objectives to their future careers, and the emphasis that should be placed on informatics content in the medical school curriculum at the University of Utah. Survey results have informed efforts to integrate informatics content into the recently revised medical school curriculum (described elsewhere).^14^

## Methods

The study was deemed exempt from the federal regulations governing human research by the University of Utah Institutional Review Board under 45 CFR 46.101(b), Category 2. Research in this category (which involves the use of educational tests, survey procedures, interview procedures or observation of public behavior) is exempt unless: (i) information obtained is recorded in such a manner that subjects can be identified, directly or through identifiers linked to the subjects; and (ii) any disclosure of the human subjects' responses outside the research could reasonably place the subjects at risk of criminal or civil liability or be damaging to the subjects' financial standing, employability or reputation.

### Survey development

Data for this cross sectional study were collected by a Web-based survey created with E-Survey software available from the Mission-Based Management Office at the University of Utah. The survey was created based on the MSOP informatics learning objectives which were designed to provide medical school graduates with a foundation to use increasingly complex information for problem solving and decision making.^7^ The MSOP Advisory Panel organized these objectives by physician roles in which informatics was thought to play a vital part: Life-long Learner, Clinician, Educator/Communicator, Researcher and Manager.^7^ The items in the survey instrument were grouped by the same physician roles. Individual survey items were created using action verbs from the cognitive domain of Bloom’s taxonomy.^15^ A single stem—How important will it be to your future career as a physician and [physician role] to: —was used to introduce the MSOP learning objectives. One or more questions based on MSOP sub-objectives were then posed to assess student perceived importance of specific abilities. For example, question #1 in the Life-long Learner question set asked, “How important will it be to your future career as a physician and Life-long Learner to: demonstrate knowledge of information resources/tools to support learning, including...a. MEDLINE and other relevant bibliographic databases? b. textbooks and reference sources? c. diagnostic expert systems? d. medical Internet resources?” Other questions in the Life-long Learner set asked students about the importance of retrieving information; filtering, evaluating and reconciling information; and exhibiting good information habits.The Clinician question set asked students about the importance of retrieving, documenting and sharing patient-specific information using a clinical information system; interpreting laboratory tests; incorporating uncertainty into clinical decision making; making critical use of decision support; formulating a treatment plan; and respecting patient and physician confidentiality. Students were asked to rate the importance of selecting and utilizing information resources for professional and patient education and employing written, electronic and oral communication in the Educator/Communicator question set. The questions in the Researcher set focused on the importance of determining what data exist relative to a clinical question or research hypothesis; executing a plan for data collection and organizing data for analysis; analyzing, interpreting and reporting findings; and appreciating the impact of information technology on basic biomedical research. In the Manager question set, students were asked to rate the importance of appreciating the role of information technology in managing the cost of medical care and its impact on individuals and society; formulating and making decisions for individuals and groups; and working effectively as an individual, in inter-professional groups and as a member of a complex health care system.

Respondents answered the questions using a five-point Likert-type scale with response options ranging from “Not at all important” (assigned a numerical value of 0) to “Extremely important” (assigned a numerical value of 4). The following question was asked at the end of each physician role question set: “Overall, how much emphasis do you think should be given to the topics covered by the [physician role] learning objectives?” Response options included “minimal” (assigned a numerical value of 0), “moderate” (assigned a numerical value of 1) and “large” (assigned a numerical value of 2) amount. The initial paper survey was pre-tested by two University of Utah medical students (one second-year and one third-year student); the online version was pre-tested by two different medical students (one second-year and one third-year student), one of whom was matriculated at the University of Utah and the other at another medical school. The survey was revised based on student feedback prior to distribution. The final survey consisted of 33 questions. However, 19 of these were question stems associated with several sub-questions, in effect increasing the total number of questions to 76. The survey required approximately 15 to 20 minutes to complete. A copy of the survey instrument may be obtained by contacting the first author.

### Study procedures

All medical students who were enrolled in the University of Utah, School of Medicine during the spring of 2008 were eligible to participate in the study. After obtaining permission from the Dean in the Office of Curriculum, an invitation to participate was distributed by the first author via e-mail to all medical students (N = 405). The e-mail explained the purpose of the survey and contained an embedded link to the survey instrument. Participation was voluntary and responses were collected anonymously (the names of the survey responders were provided to the investigators in order to send the non-responders “reminder” e-mails to complete the survey; however, the responses could not be linked to the individuals who submitted them.) Students who wished to participate were given two weeks to complete the survey. A “reminder” e-mail was sent to invitees who had not completed the survey after one week, and again 24 hours prior to the survey campaign end date. In order to maximize the response rate, two survey rounds were conducted several weeks apart, in April 2008 and May/June 2008. Respondents received a $5.00 gift card for use at campus restaurants upon survey submission as a “thank you” for their time.

### Statistical analysis

Data were analyzed using the statistical software SAS, version 9.3. Cronbach’s alpha was used to assess the internal consistency of the survey instrument. A value of 0.70 or higher was considered an acceptable level of consistency among grouped survey items.^16^ Descriptive statistics (means, frequencies and proportions) were used to address study objectives. Responses to survey items for each of the physician roles were averaged, and ANOVA was conducted to detect statistically significant differences by academic year. In contrast to the multiple items used to gauge the importance of the learning objectives, only a single survey item was used to query students about the amount of emphasis. Therefore, frequencies and proportions for the responses were calculated and the Kruskal-Wallis test was used to determine whether there were significant differences by year. A p value of ≤ 0.05 was considered statistically significant. Qualitative analysis was conducted on free-text responses to two survey items: “Are there other medical informatics learning objectives that you think should be integrated into the curriculum?” and “Additional comments.”

## Results

### Survey response rate

A total of 259 students submitted a survey for an overall response rate of 259/405 or 63.9%. The first and second rounds of survey invitations resulted in a response rate of 185/405 or 45.7% and 74/220 (the total number of students minus the 185 who had already responded to the survey) or 33.6% respectively. The overall response rates were 82/102 (80.4%), 58/96 (60.4%), 61/103 (59.2%) and 58/104 (55.8%) for first-, second-, third- and fourth-year students respectively. Data from both rounds of the study were combined in the analysis. Not every respondent answered every question on the survey. Survey data for three students were excluded from analysis for the following reasons: the student left every question blank, entered data for one question only or did not take the survey questions seriously (for example, entered zero for age). The survey analysis is based upon responses from the remaining 256 students.

### Study participants

The University of Utah School of Medicine is a state-assisted institution and the majority of participants were Utah residents. The mean age of all survey respondents was 28.0 years. Almost two-thirds of the respondents were male (64.5%). The majority of students self-reported their race and ethnicity as White, non-Hispanic (83.9%). Students were asked to indicate their intended career paths (they could select more than one option). Overall, more than two-thirds of students (67.3%) reported that they intended to enter private practice. Other selected career paths were academic medicine (44.1%), medical education (30.7%), researcher (20.1%), medical administration (11.0%), and government (6.7%). Fifteen percent of students selected the options “other” and/or “don’t know yet.”

### Reliability of the survey tool

Values for Cronbach’s alpha were 0.92 for the set of survey items related to the Life-long Learner role, 0.92 for the set of items related to the Clinician role, 0.86 for the set of items related to the Educator/Communicator role, 0.94 for the set of items related to the Researcher role and 0.94 for the set of items related to the Manager role. As the values were all greater than 0.70, the set of items for each physician role was determined to have a relatively high internal consistency. Cronbach’s alpha values of as high as 0.94 indicate that some of the individual items in the Researcher and Manager sets may have been redundant; however, as the primary focus of this study was on the information collected by the survey (and not evaluation of the survey instrument per se), all items were retained in the survey.

### Student perceived importance of learning objectives

Mean data regarding student perceived importance of the MSOP informatics learning objectives by academic year are summarized in Table 1. The learning objectives for the Clinician role received the highest mean score across all years (3.29 ± 0.47) while those for the Researcher role received the lowest overall score (2.62 ± 0.69). A statistically significant difference in the mean score by academic year was noted for the Manager role, with second-year students placing greater importance on these objectives than third-year students (t_232_ = 2.58, p = 0.01) and fourth-year students (t_232_=2.10, p=0.04). Statistically significant differences in mean scores were also noted for the Educator/Communicator and Researcher roles, with first-year students placing greater importance on these objectives than third-year students (t_213_ = 1.94, p = 0.05 and t_228_ = 2.28, p = 0.02 respectively).

**Table 1:** Mean scores for student perceived importance of the MSOP informatics learning objectives by academic year (N = 256)

Physician role	Number of items	1^st-^ year	2^nd-^ year	3^rd-^ year	4^th-^ year	All years	ANOVA	p values
		Mean (SD)	Mean (SD)	Mean (SD)	Mean (SD)	Mean (SD)		
Clinician	14	3.36 (0.45)	3.31 (0.48)	3.23 (0.46)	3.25 (0.49)	3.29 (0.47)	F_(3, 229)_=1.02	p=0.39
Life-long Learner	20	3.04 (0.56)	3.08 (0.46)	3.03 (0.53)	3.07 (0.44)	3.05 (0.51)	F_(3, 229)_=0.15	p=0.93
Manager	11	2.84 (0.71)	2.96 (0.57)^a^	2.62 (0.70)^b^	2.66 (0.78)^c^	2.78 (0.70)	F_(3, 232)_=2.82	p=0.04*
Educator/ Communicator	6	2.76 (0.74)^d^	2.74 (0.52)	2.54 (0.50)^e^	2.61 (0.66)	2.67 (0.62)	F_(3, 213)_=1.59	p=0.19
Researcher	11	2.76 (0.74)^f^	2.58 (0.64)	2.49 (0.62)^g^	2.60 (0.72)	2.62 (0.69)	F_(3, 228)_=1.83	p=0.14

*p ≤ 0.05. (a, b: t_232_ = 2.58, p = 0.01); (a, c: t_232_ = 2.10, p = 0.04); (d, e: t_213_ = 1.94, p = 0.05); (f, g: t_228_ = 2.28, p = 0.02).

Although no significant difference was seen across academic years for the Clinician role, a significant difference was noted in the mean scores for subset 10 a-c of the Clinician learning objectives, with first-year students assigning the ability to formulate a treatment plan a higher level of importance compared to third-year students (3.53± 0.52, and 3.33 ± 0.61 for first- and third-year students respectively, t_242_ = 2.13, p = 0.03). There was a significant difference in the mean scores for subset 13 a-b of the Educator/Communicator learning objectives, with third-year students assigning the ability to select and utilize information resources for professional and patient education a lower level of importance compared to second-year students (2.58 ± 0.59 and 2.89 ± 0.73 respectively, t_238_ = 2.31, p 0.02). The mean scores for subset 21 a-d of the Manager learning objectives (the ability to appreciate the role of information technology in managing the cost of medical care and its impact on individuals and society) ranged from a low of 2.53 ±0.79 for third-year medical students to a high of 2.92 ±0.65 for second-year students (t_241_ = 2.71, p = 0.01). A significant difference by academic year was also noted for subset 22 a-d of the Manager learning objectives (the ability to formulate and make decisions for individuals and groups), with mean scores of 2.83 ± 0.75, 2.92 ± 0.67, 2.60 ± 0.75, 2.53 ± 0.92, for first-, second-, third- and fourth-year students respectively (F_(3,236)_ = 3.22, p = 0.02). There were no significant differences in the mean scores by year for subsets of the Life-long Learner and Researcher objectives.

### Student desired emphasis on learning objectives

Data regarding the amount of emphasis that students felt should be placed upon the MSOP learning objectives are summarized in Table 2. Ninety-four percent of students desired a moderate or large amount of emphasis on the learning objectives associated with the Clinician role. Ninety-two percent of students reported that objectives for the Life-long Learner role merited a moderate or large emphasis in the curriculum; 83% thought the content associated with the Manager role should receive this level of emphasis. The percentage of students who assigned a moderate or large degree of emphasis to the objectives for the Researcher and the Educator/Communicator roles were 64% and 66% respectively. A significant difference by academic year was noted for the amount of emphasis that should be given to learning objectives associated with the Life-long Learner role (χ^2^(3) = 8.615, p = 0.03), with higher proportions of first-year students assigning “minimal” or “moderate” emphasis to this set of objectives compared to students in other years. No significant differences by year were noted for the other physician roles.

**Table 2:** Number and percent of students assigning minimal, moderate or large degree of emphasis to MSOP informatics learning objectives by academic year (N = 256)

Physician role	Amount of Emphasis	1^st-^ year	2^nd-^ year	3^rd-^ year	4^th-^ year	All years	Kruskal-Wallis	p value
		n (%)	n (%)	n (%)	n (%)	N (%)		
Clinician	Minimal	4 (5)	4 (7)	5 (9)	3 (6)	16 (7)	χ^2^(3)=1.025	p=0.80
	Moderate	48 (59)	28 (49)	32 (54)	26 (51)	134 (54)		
	Large	29 (36)	25 (44)	22 (37)	22 (43)	98 (40)		
Life-long Learner	Minimal	12 (15)	4 (7)	3 (5)	3 (6)	22 (9)	χ^2^(3)=8.615	p=0.03*
	Moderate	51 (63)	30 (54)	34 (58)	30 (58)	145 (59)		
	Large	18 (22)	22 (39)	22 (37)	19 (37)	81 (33)		
Manager	Minimal	17 (21)	8 (14)	7 (12)	10 (21)	42 (17)	χ^2^(3)=5.152	p=0.16
	Moderate	48 (59)	27 (48)	40 (66)	26 (54)	141 (57)		
	Large	16 (20)	21 (38)	14 (23)	12 (25)	63 (26)		
Researcher	Minimal	30 (37)	20 (36)	21 (34)	17 (33)	88 (35)	χ^2^(3)=0.424	p=0.94
	Moderate	40 (49)	26 (46)	32 (53)	25 (49)	123 (49)		
	Large	11 (14)	10 (18)	8 (13)	9 (18)	38 (15)		
Educator/ Communicator	Minimal	31 (38)	18 (32)	20 (33)	15 (29)	84 (34)	χ^2^(3)=1.297	p=0.73
	Moderate	40 (49)	31 (55)	36 (59)	28 (55)	135 (54)		
	Large	10 (12)	7 (13)	5 (8)	8 (16)	30 (12)		

*p ≤ 0.05.

### Gender differences

There were no significant differences by gender in student-perceived importance for the set of learning objectives associated with the Clinician, Educator/Communicator, Manager and Researcher physician roles. However, statistically significant differences by gender were noted for the role of Life-long Learner.

The overall score for the set of Life-long Learner objectives was significantly higher for female compared to male students (3.19 ± 0.47 and 3.05 ± 0.43 respectively, t_249_=2.15, p = 0.03). Female medical students rated the ability to retrieve information—including the ability to perform database searches using logical (Boolean) operators, refine search strategies to improve the relevance and completeness of retrieved items, use a standard bibliographic application to download citations and organize them into a personal database and identify and acquire full-text electronic documents available from the WWW or a local "virtual" library—higher than their male classmates (3.11 ± 0.63 and 2.93 ± 0.61, respectively, t_249_=2.10, p=0.04). There were no significant differences by gender regarding the amount of emphasis that should be placed on the learning objectives for each physician role.

### Student comments

Thirty-six students from all four academic years provided comments regarding other informatics objectives that should be integrated into the curriculum. A higher percentage of first-year students provided comments compared to students in other years (16/36 or 44.4%, 7/36 or 19.4%, 8/36 or 22.2% and 5/36 or 13.9% for first-, second-, third- and fourth-year students respectively). Perhaps not surprisingly, first-year students expressed concerns about information overload. First-year students also desired training on portable digital applications and smart phones. Students from all years acknowledged the importance of training in information retrieval and management, use of tools/technology and clinical applications/electronic medical record systems.Sixteen students provided additional comments. Of those providing comments, 9/16 or 56.3%, 3/16 or 18.8% and 4/16 or 25.0% were first-, second- and third-year students respectively. Several common themes emerged (Table 3). Some first-year students were skeptical about the applicability of informatics to their careers; others felt the learning objectives were important but would be difficult to integrate. Ideas were provided regarding informatics content, delivery format and timing, including the suggestion that third- and fourth-year students might benefit most from informatics instruction due to their clinical experience.

**Table 3:** Sample student responses to the survey item “additional comments”

Theme	Student comment
Skepticism about applicability of informatics to future career	*“...We don’t need to learn more things we’re not going to use in 20 years.”*[1^st^-year student] *“I don’t want to learn anything but how to be a good clinician...”* [1^st^ -year student]
Importance of informatics learning objectives	*“...many of these objectives are important...”* [2^nd^ - year student] *“These are important issues...”* [2^nd^ - year student]
Difficulty of teaching/integrating informatics content	*“...integrating...will be difficult because it does not necessarily fit within the current structure and would be difficult to transform into a classroom setting...”* [3^rd^ - year student] *“Medical informatics is difficult to teach...”* [3^rd^- year student]

## Discussion

Demographic characteristics of the survey respondents reflected those of the larger medical student population. Overall, the Cronbach's alpha-coefficients of internal reliability (standardized) were all above the acceptable standard of 0.70, indicating a high internal consistency of the survey.The survey response rate declined with advancing academic year. Third- and fourth-year students were likely participating in clinical rotations at the time the survey was distributed. As a result, they may not have felt they had the time to complete a voluntary survey, unlike first-year students who were spending time in the classroom and may have had a greater interest in completing a survey asking for their input about learning content.Medical student perception regarding the importance of the MSOP informatics learning objectives to their future careers ranged from “somewhat” to “very” important. While none were thought to be “not at all” important, no set of learning objectives received a rating of “extremely” important. Perhaps not surprisingly, the Clinician learning objectives received the highest overall rating, followed in descending order by Life-long Learner, Manager, Educator/Communicator and finally Researcher.

It is unclear why students rated the Researcher role as least important. This physician role may have been misunderstood by the students, contributing to its low ratings. As defined by the MSOP report, this role involves the ability to define the case mix of your practice, monitor trends in disease incidence and use calculations such as the predictive value positive to determine which lab tests to order and when to order them.^7^ The medical students completing the survey may have envisioned a non-clinical role for the physician as researcher, for example as someone who exclusively conducts scientific experiments in a laboratory and is not involved in the care of patients. Interestingly, even students enrolled in MD-PhD training programs disagreed with the current working definition of a physician-scientist according to a recent survey.^17^There were significant differences in student ratings by academic year, with third-year students consistently assigning lower importance ratings to the group of learning objectives associated with the Educator/Communicator, Researcher and Manager roles compared to students in some other years. Making the transition from the classroom to patient care in the third year involves significant learning challenges^18^ and may be stressful.^19^ The third-year students’ recent entry into the clinical arena may have contributed to the increased importance of the Clinician role in their eyes, and to a shorter-term view of other skills that may be important later in their careers.While the results are interesting, it is difficult to explain why the female students who participated in the present study ascribed greater importance in terms of their future careers to the learning objectives associated with the Life-long Learner role than the male students. This finding warrants further study to determine if the results are replicable. In a study of Jefferson Medical College graduates in which the predictors and outcomes of life-long learning were investigated, gender differences on the life-long learning scores were negligible.^20^Student perception regarding the degree of emphasis that should be placed on content related to the learning objectives also varied by physician role. A higher proportion of students believed that a moderate-to-large amount of emphasis was appropriate for the Clinician, Life-long Learner and Manager roles compared to the Researcher and Educator/Communicator roles, consistent with the lower ratings the latter roles received for perceived importance. A significant difference was noted between the first-year students and those in later years regarding the amount of emphasis that should be given to the Life-long Learner objectives. Students at the beginning of their academic careers may not yet understand the central role that life-long learning skills will play in their future careers. A higher proportion of fourth-year students assigned the content associated with the Educator/Communicator role a large amount of emphasis, perhaps placing greater value on these skills as a result of interaction with patients during the clinical years.To our knowledge, this is the first study (MEDLINE search) that has queried medical students about the importance of informatics learning objectives in terms of role development. Survey responses revealed that informatics content is desired by medical students at the University of Utah. Faculty from the University’s Department of Biomedical Informatics have recently partnered with health sciences librarians from the University’s Spencer S. Eccles Health Sciences Library to form the Biomedical Information and Informatics (BMII) Thread, one of several content areas being woven into the revised medical school curriculum that was implemented in August 2009. Efforts are ongoing to incorporate BMII Thread content into all four years of the curriculum.For example, additional opportunities have been created within the curriculum to address informatics learning objectives associated with the Clinician, Life-long Learner and Manager physician roles which received the highest rankings in terms of both importance and amount of emphasis. New “mini” lectures and related exercises have been developed to provide first-year students with hands-on opportunities for information retrieval, such as using PubMed to answer clinical questions or an electronic health record training environment to locate clinical data for a fictitious patient. An introduction to the concept of clinical decision support has been integrated into the second year, and students learn how informatics applications may enhance patient safety in year three. A session outlining the critical role of informatics in the process of quality improvement will be conducted in the coming academic year with fourth-year students. Despite the fact that the Researcher role was assigned only a minimal to moderate emphasis score by the students, a new requirement to complete a hypothesis-based scholarly project prior to graduation has been implemented by the School of Medicine.Others are calling for biomedical informatics to play a greater role in the education of medical students, residents and fellows.^21^^-^^23^ Efforts to weave informatics content into curricula have been undertaken by other medical schools, including one with a significant focus on informatics.^24^ Residency training programs in several clinical specialties are also integrating informatics into their curricula.^25^^-^^28^ The Board of Directors for the American Medical Informatics Association has approved program requirements for fellowship education in clinical informatics.^29^ Recently, the American Board of Medical Specialties approved clinical informatics as a medical subspecialty, with board certification in clinical informatics granted by the American Board of Preventive Medicine.^30^ As a result of these increased educational opportunities in biomedical informatics, physicians-in-training will develop the expertise needed to practice medicine in a 21^st^ century health care system.There are several limitations of the present study. It is a cross-sectional survey and, as such, offers only a snapshot of student opinion at a single point in time. The study employed a convenience sample which may have introduced a source of bias. For example, the non-responders may have believed that informatics was less important to their future careers than the responders. Furthermore, the survey results may not be generalizable to medical schools with different student populations or curriculum models.

## Conclusions

Medical students at the University of Utah reported that acquiring skills related to biomedical informatics will be important to their future careers as physicians. Learning objectives for the physician roles of Clinician, Life-long Learner and Manager received higher ratings than the Educator/Communicator and Researcher roles in terms of both perceived importance and the amount of emphasis these content areas should receive in the curriculum. Objectives associated with the physician role of Clinician received the highest overall rating. Student ratings regarding the importance of the informatics learning objectives and the amount of emphasis these should receive varied by academic year. While acknowledging the importance of informatics training in undergraduate medical education, students expressed concerns that this topic might be a difficult one to integrate into the curriculum. Survey data are being used to inform efforts to integrate biomedical informatics content into the medical school curriculum. Study findings may assist other medical schools seeking to incorporate similar content into their curricula.

## 

### Conflict of Interest

The authors declare that they have no conflict of interest.
